# Angiotensin-Converting Enzyme (ACE) Insertion/Deletion (I/D) Polymorphism as a Conjoint Regulator of Coagulation, Fibrinolytic, and RAAS Pathway in Infertility and Associated Pregnancy Complications

**DOI:** 10.1155/2022/1695769

**Published:** 2022-11-29

**Authors:** Sunil Thakur, Vaishnavi Sharma, Dipneet Kaur, Pulakes Purkait

**Affiliations:** ^1^Origin LIFE Healthcare Solutions & Research Centre LLP, Chandigarh PIN-160036, India; ^2^Postgraduate Government College for Girls, Sector-42, Chandigarh, India

## Abstract

Despite the increase in assisted reproductive technologies, the high rates of infertility and pregnancy complications are a major concern to infertility specialists worldwide. Infertility may be attributed to pregnancy complications like thrombophilia, preeclampsia and fibrin-induced recurrent pregnancy loss (RPL). Renin-angiotensin-aldosterone system (RAAS) directly or indirectly causes preeclampsia and thrombophilia through the fibrinolytic pathway that ultimately leads to RPL or infertility. The underlying mechanisms of this interaction are still unclear. The present comprehensive review is intended to demonstrate the role and interaction of RAAS and fibrinolytic pathways in pregnancy complications. How this interaction can induce pregnancy complications, and ultimately infertility, is also discussed in the light of current evidence. This study also presents common markers that link RAAS and fibrinolytic processes in developing thrombophilia, preeclampsia and RPL. The common link in these pathways is ACE gene I/D polymorphism. Apart from ACE, PAI-1, VIIa, XIIa, AT1R, AT1AA, and TF are common molecules that can delineate the underlying causes of pregnancy complications and infertility.

## 1. Introduction

Infertility is the inability of a couple to conceive despite regular, unprotected sexual intercourse over twelve months. This period of inability to conceive might be age-dependent [1]. In women over 35 years, it is reduced to six months as advanced age has a greater risk of infertility [2]. Broadly, a failure of delivering a viable pregnancy is infertility. Based on the efficiency of parity and gravidity, infertility can be primary and secondary. Primary infertility is an inability of a couple to conceive, while secondary infertility is having at least one term pregnancy or miscarriage [2]. Secondary infertility is the commonest form of infertility among females. It is influenced by reproductive tract diseases (hypogonadotropic hypogonadism, hyperprolactinemia, disorders of ciliary function, cystic fibrosis, infections, and systemic diseases), reproductive tract infections, advanced age, and lifestyle-related factors. Other factors, such as the decline in ovarian reserve, endometriosis, polycystic ovary syndrome (PCOS), uterine fibroids, and endometrial polyps, can also act as infertility inducers in females [1]. Infertility may be attributed to pregnancy complications like thrombophilia and preeclampsia (PE). Thrombophilia and PE are related to fibrin-induced recurrent pregnancy loss (RPL) [3].

The renin-angiotensin-aldosterone system (RAAS) is one of the most active pathways for homeostasis, blood pressure regulation, and water balance in the body. Previous studies comprehensively described the function, regulation, and therapeutic role of RAAS in cardiovascular and renal diseases [4, 5]. RAAS pathway is one of the significant blood pressure regulators of the body that directly or indirectly controls the fibrinolytic pathway [6]. The effect of RAAS and fibrinolytic interface can be seen on the prevalence of PE and thrombophilia that ultimately leads to RPL or infertility [7]. Although RAAS-fibrinolysis interaction impacts pregnancy enormously, the underlying mechanisms are still unclear. The present comprehensive review is intended to demonstrate the role and interaction of RAAS and fibrinolytic pathways in pregnancy complications. How this interaction can induce pregnancy complications, and ultimately, infertility is also discussed in the light of current evidence. This study also presents common markers that link RAAS and fibrinolytic processes in developing thrombophilia, PE, and RPL.

## 2. Epidemiology of Infertility

The literature shows that infertility is a problem that affects either of the partners. However, female infertility is considered a massive problem in developing countries. It causes more than 50% of the infertility cases, while the remaining is associated with sperm disorder or unknown factors [1]. Today, one in seven couples has been estimated to be infertile globally. This figure is more drastic in developing countries where infertility prevalence is reduced to one in four couples [8]. Approximately eighty million couples are experiencing involuntary infertility worldwide [9]. Asia (South and Central), Africa (except south and east), and Europe (Central and Eastern) exhibit higher infertility rates, reaching as high as 30% [1]. Worldwide, primary infertility ranges from 3 to over 30%, while secondary infertility is double primary infertility [9]. According to a study, for each increase of 9% in the proportion of women aged 45-49 with no children, fertility decreases by one birth [10]. While talking about the Indian subcontinent and India, the rate of infertility showed an increase despite the increase in assisted reproductive technologies (ARTs). A study showed that 2015-2016 infertility rates are remarkably higher than 2005-2006 in India [10]. Approximately, 15-20 million out of 60–80 million infertile couples are in India alone with region-specific prevalence. Southern Indian states are most commonly affected. Urban women are more prone to infertility than their rural counterparts due to environmental and lifestyle differences. Vegetarian diet, overweight, obese, and thyroid patients are strongly associated with infertility [10–12]. Ganguly et al. [12] found that education and standard of living (SOL) are also inversely related to infertility. The plausible reason for this relationship was the awareness and increased access to expensive treatments among working women. Urgent actions are needed to handle the current magnitude of the infertility problem as most of the factors causing it are avoidable.

## 3. Factors Affecting Female Infertility

Infertility is majorly influenced by diseases related to the reproductive tract, the age of couples, and lifestyle-related factors [1]. Clinical conditions such as the decline in ovarian reserve, endometriosis, PCOS, uterine fibroids, and endometrial polyps also act as infertility inducers [1]. The demographic factors such as higher educational level, employment, staying in nuclear families, and high socioeconomic conditions have also been significantly associated with infertility. Rapid urbanization, elevated SOL, and the rise in the education status of women are suggested as some of the leading causes of infertility [10]. Apart from the above-listed environmental and lifestyle factors affecting women's fertility status, the interaction of genetic components with environmental stresses cannot be ignored. The genetics of women is affected by infectious or parasitic diseases, lifestyle, stress, postponing parenthood, and obesity which are considered essential determinants of infertility. The factors influencing infertility can broadly be attributed to ovarian factors, tubal and peritoneal factors, anomalies, advanced ages (over 35 years), hormonal disorders, habits, genetic factors, medical conditions, and lifestyle.

### 3.1. Hormonal Factors

For a healthy reproductive life of women, a proper hormonal balance is very much essential. Any imbalance in hormonal regulation can cause infertility and related diseases. Hormonal balance is vital for efficient reproductive cycles and associated processes such as ovulation and overall conception systems [2]. In maintaining hormonal balance in the reproductive tract, the hypothalamus is the master regulator that releases gonadotropin. Gonadotropin directly or indirectly affects the pituitary gland, ovaries, thyroid, and mammary gland [13, 14]. Thus, modified chemical signals from the hypothalamus can cause hormonal abnormalities that result in infertility. Hypothyroidism, PCOS, and hyperprolactinemia are diseases caused by hormonal imbalance [13, 15]. Ovulation, menstruation, implantation, and pregnancy are significant events in female reproductive function controlled by hormonal and inflammatory processes. Increasing evidence shows that hormonal aberrations and a hyperinflammatory state may derange the immune-endocrine cross-talk among endometrium, myometrium, and cervix, and between the decidua and trophoblast predisposing to pregnancy complications.

### 3.2. Lifestyle Factors

Lifestyle choices play a pivotal role in the fertility of an individual. Many factors like alcohol intake, tobacco smoking, dietary restriction, overexercise, and obesity contribute to infertility [1]. Poor diet intake, calorie restriction, and overexercise lead to an increase in the frequency of ovulation and poor endometrial development [13]. Obesity is reported as one of the leading causes of ovarian dysfunction. An increase in weight is directly proportional to the production of estrogen, which limits the chances of getting pregnant as our body interprets it as birth control [2, 16]. High estrogen levels make it difficult for overweight females to ovulate and conceive even after infertility care. Lifestyle choices like smoking affect infertility to the worst extent. Smoking products (e.g., cigarettes) contain nicotine which interferes with reproductive processes such as estrogen synthesis, embryo transport, endometrial receptivity, endometrial angiogenesis, uterine blood flow, and the uterine myometrium [17]. Hence, lifestyle choices make a difference in a viable or nonviable and healthy or unhealthy pregnancy. The absence of a healthy lifestyle can lead to pregnancy complications such as thrombophilia, PE, and RPL [18–20].

### 3.3. Genetic Factors

Apart from the above listed modifiable causes (mostly lifestyle-related), the nonmodifiable causes (primarily genetic) can also alter the pregnancy outcomes. Gene-environment and gene-gene interaction that facilitates the normal functioning of the female reproductive system are also essential regulators of pregnancy. Any aberrations in genetic factors can lead to infertility. Genetic factors are responsible for at least 35% of all infertility cases [21]. These factors commonly affect the development of ovaries, oocytes maturation, and fertilization competence. Genetic abnormalities such as chromosome abnormalities, submicroscopic chromosome deletion and duplications, and gene sequence variation are the major genetic causes of female infertility [22]. Whole-genome sequencing revealed that even single gene causes could explain infertility and pregnancy complications [23]. However, these complications are often challenging due to their multitissue and multiorganism nature. Research in the genetic and gene regulatory causes of pregnancy complications has the potential to revolutionize our understanding of healthy and complicated pregnancies.

#### 3.3.1. The Angiotensin-Converting Enzyme (ACE)

**Figure 1:**
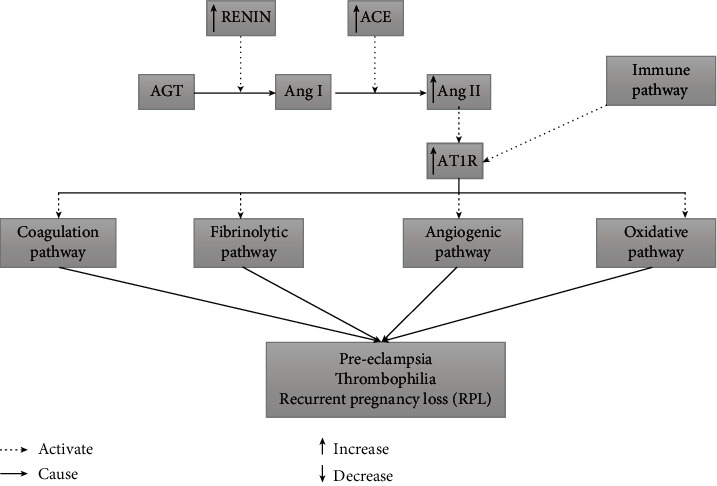
Interaction of RAAS pathway with other pathways to cause pregnancy complications.

The RAAS regulates blood pressure and water balance, which play a role in developing many cardiovascular disorders, including PE, thrombophilia, RPL, and others [7]. Local RAAS in the pancreas and the placenta are involved in physiological and pathophysiological processes in pregnancy. Angiotensin I-converting enzyme (ACE) is a pivotal component of the renin-angiotensin-aldosterone system. It is a zinc metallopeptidase that cleaves angiotensin I to form vasoconstrictor angiotensin II (Figure 1). When blood volume is low, ACE hydrolyzes angiotensin I to angiotensin II, a potent vasoconstrictor, increasing blood pressure.

#### 3.3.2. ACE Gene and I/D Polymorphism

One of the vital components of RAAS, the ACE gene, is located on chromosome 17q23, and it consists of 26 exons and 25 introns [24]. The gene encodes two isoforms of ACE: the large (170 kDa) somatic form (sACE), which is expressed in somatic tissue, and the small (100 kDa) testicular form (tACE) or germinal ACE (gACE), expressed in germinal cells in the testes [25] There are more than 160 ACE gene polymorphisms, according to the National Center for Biotechnology Information (NCBI) records, dominated by single nucleotide polymorphisms (SNPs). About 34 are located in coding regions; 18 are missense mutations [25]. Polymorphisms of the ACE gene are associated with some vascular diseases [26]. An immense number of studies have been published that investigated the association between the I/D polymorphism and clinical outcomes of diseases, covering the aspects like symptoms and manifestations, the efficacy of drugs and therapies, interaction with other genetic or environmental factors, recovery rates, disease progression, and survival.

#### 3.3.3. ACE I/D Polymorphism and Pregnancy Complications

ACE I/D-polymorphism is the most studied and correlated polymorphism of the ACE gene consisting of the insertion or deletion (I/D) of a 287-bp fragment in intron 16. It is associated with control of circulating ACE levels and accounted for approximately half (47%) of the observed variance in ACE levels [27]. The ACE I/D polymorphism plays a vital role in female infertility. The DD genotype has previously been shown to be a risk factor in pregnancies complicated by preeclampsia [28], intrauterine growth restriction (IUGR) [29], and recurrent spontaneous miscarriages [30], all of which have a common factor of impaired placentation [31]. In addition, the DD genotype has been reported to influence the regulation of fibrinolytic systems and inflammation [32]. This system is critical in the development of thrombophilia. Some investigators reported an association between ACE gene DD genotype and a high preeclampsia risk [7]. The ACE ID and DD genotypes were also associated with a higher risk of endometrial tumors [33]. The ACE I/D polymorphism predisposes women to PCOS at an early age and is significantly associated with acanthosis, a marker of insulin resistance [34]. However, ACE I/D polymorphism is associated with pregnancy-related complications and infertility through multiple pathways; the actual mechanism of ACE activity in this effect is still unclear. This review explores the interlinking of thrombophilia, PE, and RPL pathways through the ACE gene and associated mechanisms.

## 4. Thrombophilia

### 4.1. Pathophysiology of Thrombophilia

The balance between maternal coagulation and fibrinolysis is vital in maintaining a stable and healthy pregnancy. It prevents excess fibrin deposition in placental vessels and intervillous spaces, secures fibrin polymerization, and stabilizes the placental basal plate [35]. Women with thrombophilic defects are at increased risk for pregnancy-associated thromboembolism and other vascular complications, such as PE [36] and fetal loss [37]. Pregnancy is a hypercoagulation state which is presented with increased coagulants (factors VII-XII and fibrinogen), decreased anticoagulants (protein C and protein S), and reduced fibrinolytic activities [38]. Thrombophilia, whether acquired or inherited, is also a hypercoagulation state. The hypercoagulation states were shown to be associated with recurrent pregnancy loss, intrauterine fetal growth restriction (IUGR), PE, and venous thromboembolism [39].

Further, in the coagulation process, Factor XIII acts as a resistance to thrombus formation and has been associated with antiphospholipid syndrome (APS) [40]. APS is associated with adverse pregnancy outcomes [41, 42]. APS can cause venous, arterial, and small vessel thrombosis, pregnancy loss, preterm delivery, PE, and placental insufficiency [3, 43]. Inherited thrombophilia is also associated with pregnancy complications. Factor V Leiden, prothrombin mutation, antithrombin, protein C and S deficiency have been studied and associated with recurrent miscarriage [44].

### 4.2. Genetics of Thrombophilia

**Figure 2:**
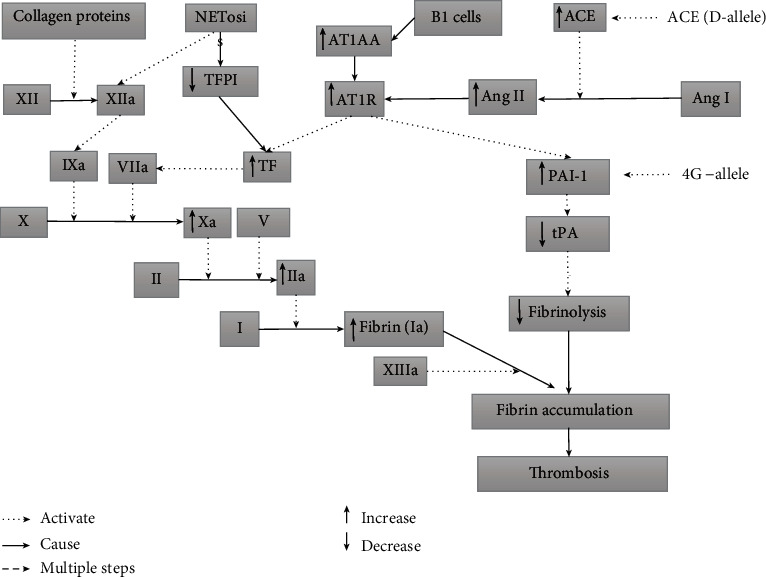
Interaction of RAAS, coagulation, immune, and fibrinolytic pathways in thrombophilia.

The fibrinolytic pathway mainly regulates thrombophilia so that blood clotting can be controlled. The ACE gene also has a physiological function in the fibrinolysis pathway as it regulates the concentrations of plasminogen activator inhibitor-1 (PAI-1), an essential determinant in the control of the fibrinolytic process [38]. In the presence of the DD genotype, the increased ACE enzyme activity enhances the production of angiotensin II from angiotensin I, which is associated with high circulating PAI-1. The high levels of PAI-1 result in the inhibition of fibrinolysis. The genetic variants of PAI-1(4G/5G) and ACE gene (I/D polymorphism) were associated with the placental formation and trophoblast invasion [27, 35]. AGT and AT1R are two other genes involved in fibrinolysis, platelet aggregation, and blood clotting at the implantation site [45]. Angiotensin II also elevates PAI-1 levels, which results in a decrease in fibrinolytic activity. Previous studies have shown that the plasma level of angiotensin II is closely associated with ACE gene polymorphisms [46, 47]. ACE I/D gene polymorphism has been described as an independent risk factor for thrombotic diseases [24]. To modify trophoblast cells and to prevent haemorrhage during placentation, PAI-1 is upregulated during implantation. The PAI-1 (-675 4G/5G) allele is associated with gene overexpression related to hypofibrinolysis and thrombotic complications for women during early pregnancy [48, 49]. Hypofibrinolysis, caused by the 4G/4G polymorphism of the PAI-1 gene, appears to be a significant independent factor for pregnancy complications, including PE, intrauterine growth retardation, placental abruption and stillbirth, and probably acts through thrombotic induction of placental insufficiency. The combined deleterious effect of the ACE D allele and the PAI-1 4G allele makes women susceptible to miscarriage owing to the link between the RAAS and fibrinolysis (Figure 2). Another pivotal gene that is most studied in thrombophilia is Factor V of the coagulation pathway. This factor helps in the conversion of prothrombin to thrombin. Protein C and S degrade FV and FVIII into inactivated forms, representing a key feedback inhibition mechanism of the prothrombin convertase in coagulation and hemostasis [45].

### 4.3. Mechanism of Thrombophilia Occurrence

The formation or accumulation of fibrin in the plasma results in blood clotting through the coagulation pathway. Excess fibrin causes thrombophilia, which leads to other pregnancy-related complications or diseases [36]. Two pathways initiate the accumulation of fibrin. The fibrinolytic pathway is linked to the RAAS pathway to cause fibrin accumulation [50]. When renin is released into the bloodstream during the RAAS pathway, it hydrolyses AGT secreted by the liver into Ang I. Ang I then converted to Ang II, a potent vasoconstrictor, with the help of the ACE enzyme. Enhanced ACE enzymatic activity also inactivates vasodilator angiotensin-(1-7). Ang II further binds with AT1R and AT2R receptors and exerts its effects. Enhanced ACE activity and Ang II-AT1R binding result in the activation of PAI-1 and its mRNA expression in tissue [51, 52]. PAI-1 is related to inhibiting a tissue plasminogen activator (tPA) that reduces fibrinolysis, resulting in fibrin formation and accumulation [53]. Maternal autoantibody (AT1-AA) can also activate AT1 receptors that account for increased PAI-1 and shallow trophoblast invasion in PE [54].

The intrinsic pathway is also demonstrated to have an effect that results in thrombophilia. In the intrinsic pathway, Factor XII is converted to XIIa with the help of collagen fibrils of various origins and initiated a cascade shown in Figure 2. The vital step of this cascade is the conversion of X to Xa. Factor Xa is formed with the help of IXa and VIIa. VIIa comes from the conversion of VII with the help of tissue factor (TF) mRNA (which is formed when Ang II binds with AT1R) [55, 56]. Xa is then led to thrombin formation, which ultimately results in fibrin accumulation with the help of XIIIa. Fibrin accumulation was also initiated by neutrophil extracellular traps (NETs). Activated NETs cleave endogenous anticoagulant TFPI (Tissue Factor Pathway Inhibitor) and initiate the contact pathway [57, 58]. NETs also activate FXII due to their polyanion properties. NETs stimulate platelets via histones H3 and H4 and binding with TF and promote the extrinsic pathway of coagulation [59]. In veins, myeloid cells also start fibrin formation upon activation. Activated myeloid cells bring in B1 cells that produce APA. APA binds with prothrombin and activates monocytes and platelets. Monocytes and platelets, in turn, deliver TF to initiate the extrinsic coagulation pathway [59, 60]. In sum, the tissue factor (TF) seems to be a connecting link between extrinsic, intrinsic, and immune pathways in thrombophilia. In future studies, its potential as a thrombophilia marker can be exploited in therapeutics.

## 5. Preeclampsia (PE)

### 5.1. Pathophysiology of PE

PE is characterized by high blood pressure, the onset of proteinuria usually after the 20th week of gestation, and new-onset hypertension in the absence of proteinuria combined with hematological complications, renal insufficiency, impaired liver function, and neurological symptoms [61]. The significant risk factors for PE development are history of PE, chronic hypertension, pregestational diabetes mellitus, antiphospholipid syndrome, and obesity [62]. The role of the human placenta in the viability of pregnancy to the term is vital. It has been reported that uteroplacental blood circulation is regulated by locally present RAAS [63]. The dysregulation (up or down) of placental RAAS could be detrimental and induce clinical pregnancy complications (such as pregnancy-induced hypertension and PE). As earlier described in this review, RAAS also plays a central role in pregnancy complications via blood pressure and water balance regulation. Clinical and pathological studies suggest that the placenta, through RAAS, is central to the pathogenesis of PE [7].

In pregnant women, there are marked increases in plasma renin concentrations, renin activity, renin substrate, angiotensin II, and aldosterone [64]. Enhanced ACE enzyme activity could lead to impaired production of biologically active angiotensin II. Further, impaired progesterone levels during pregnancy are also detrimental. Both Ang II and progesterone increase the destruction of platelets, leading to the inability to reduce angiotensin II during pregnancy [65]. Enhanced Ang II induces pregnancy-related hypertension leading to PE and further pregnancy complications [66].

### 5.2. Genetics of PE

The PE is mainly regulated by genes of the RAAS pathway, especially by the ACE gene. The ACE enzyme is involved in the conversion of Ang I to Ang II. As described previously, Ang II is a potential vasoconstrictor that leads to pregnancy-induced hypertension, one of the significant symptoms of PE. ACE also controls the activity of vasodilator angiotensin-(1-7). The decreased plasma Ang-(1-7) levels may be related to the increased ACE activity in pregnant preeclamptic women. A study demonstrated the role of ACE DD genotype in susceptibility to PE [27]. Thus, the ACE gene seems to be the most critical in the development of PE. The ACE activity increase, associated with high local AGT expression, also leads to elevated local angiotensin II levels [7]. Ang II, AGT, and AT1R are other RAAS genes directly associated with blood pressure regulation in preeclamptic women. In preeclamptic women, increased AGT expression, upregulated AT1 receptor subtypes mRNA expression, and increased responsiveness to angiotensin II has been shown [63].

### 5.3. Mechanism of PE Occurrence

**Figure 3:**
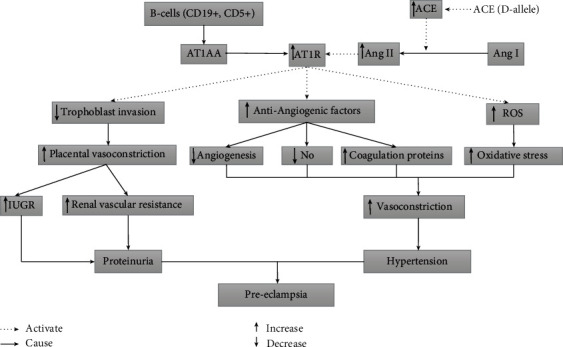
Interaction of RAAS, immune, and coagulation pathways in preeclampsia.

In the human placenta, RAAS controls the pathophysiological regulation of uteroplacental blood circulation. Decrease in arterial blood pressure leads to the activation of RAAS, wherein renin is released into the bloodstream and hydrolyses AGT secreted by the liver into Ang I. ACE converts Ang I to Ang II and also inactivate the vasodilator angiotensin-(1-7) (Ang-(1-7)) through rapid hydrolysis [7]. Ang II exerts its effect by binding to angiotensin II type 1 receptor (AT1R) and angiotensin II type 2- receptor (AT2Rs). Enhanced ACE activity and the binding of Ang II to its receptor AT1R are related to complications in PE [67]. Further, in PE women, the occurrence of angiotensin II type 1 receptor autoantibody (AT1-AA), an agonistic antibody, was also seen [68]. AT1-AA exerts a similar effect as that of Ang II, i.e., binding and activation of AT1R. AT1-AA is produced by mature B cells (CD19 (+) CD5 (+)) [69]. This AT1-AA, through multiple studies, has been shown to play a significant role in the pathology and possible genesis of PE. AT1-AAs can contribute to the impairment of the placenta and fetal development due to increased placental vasoconstriction. AT1-AAs cause an increase in blood pressure and other factors associated with PE [70]. The binding of Ang II and angiotensin II type 1 receptor autoantibody (AT1-AA) with AT1R leads to shallow trophoblast invasion of the uterine spiral arterioles, excess placental secretion of antiangiogenic factors (sFlt1, sEng) into maternal circulation and creates an imbalance between ROS generating enzymes and antioxidants [71]. Soluble fms like tyrosine kinase 1(sFlt1), soluble endoglin (sEng) production in PE leads to a decrease in nitric oxide production, impaired angiogenesis, and release of procoagulant proteins [62, 72]. All above mentioned steps result in hypertension and proteinuria, which are the systemic manifestations of PE (Figure 3). The exact cause of PE is still unknown, but RAAS pathway (especially ACE I/D polymorphism), immune pathway (AT1-AA), and antiangiogenic factors (such as sFlt1 and sEng) can be targeted in future studies to ascertain the causes of PE.

## 6. Recurrent Pregnancy Loss (RPL)

### 6.1. Pathophysiology of RPL

Recurrent pregnancy loss (RPL) is defined as three or more consecutive miscarriages before 20 weeks of pregnancy. The known etiologic factors for RPL include parental chromosome abnormalities, endocrinological disorders, hereditary thrombophilia, immunologic factors, male-factors, PE, and environmental factors [24, 48, 73]. In pregnancy, women are in a hypercoagulable state, which may impair placental flow and fetal growth. As earlier discussed in this review, hypercoagulation states predispose women to develop venous thrombosis at the challenge of delivery. In addition, the RAAS pathway is the most critical in precauses of RPL, i.e., PE and thrombophilia. As ACE and other enzymes of this pathway are associated with degenerated hemostasis, excess fibrin accumulates in spiral arteries and platelet aggregation [74, 75]. All these factors could lead to pregnancy loss. The D allele was also associated with a high serum ACE, which enhances the formation of angiotensin II, thus increasing the risk of thrombotic episodes and PE [76] and, ultimately, RPL [77].

### 6.2. Genetics of RPL

The RAAS pathway and its associated genes are the ultimate regulators of RPL through control of thrombophilia and PE. The ACE gene is a key regulator in the modulation of vascular homeostasis, inflammation, and angiogenesis. Based on its biological functions, the ACE gene's insertion/deletion (I/D) polymorphism is seen as a candidate locus for RPL [76]. The increased risk of RPL is attributed to ACE I/D polymorphism with ethnic differences [78]. ACE I/D polymorphism is delineated as a risk factor for RPL in Caucasian and West-Asian populations but not in East-Asians [24]. The ACE gene is related to plasminogen activator inhibitor-1 (PAI-1) activity, a key regulator in embryo implantation [73]. The ACE I/D polymorphism regulates ACE enzyme activity and its levels in plasma and tissues. The ACE D- allele has higher serum ACE activity than I- allele [78]. A study has shown that the ACE D allele could increase PAI-1 and Ang II, increasing the risk of thrombotic events [24]. All these events may lead to RPL.

### 6.3. Mechanism of RPL Occurrence

RPL is mainly attributed to thrombophilia, found in 40-50% of cases [77]. Thrombophilia might cause the formation of microthrombosis at the site of implantation and result in RPLs [79] PAI-1 is positively associated with spontaneous abortions and RPL through hypofibrinolysis [48]. A meta-analysis [77] in 4306 cases and 3076 controls showed an association of that PAI-1 (4G/5G) polymorphism with the risk of RPL. It was shown in Figure 2 that how PAI-1 develops thrombophilia. PAI-1 is also a key regulator of embryo implantation [73]. The activity of PAI-1 is regulated by the ACE gene of RAAS (mechanism described earlier). A meta-analysis done in 2013 showed that PAI-1 and ACE genes are associated with the risk of RPL [80]. The DD genotype of ACE is previously associated with RPL. Women with DD genotype have a 72% elevated risk of RPL development [78].

## 7. Discussion

The ACE I/D polymorphism is one significant polymorphism affecting plasma ACE levels. The genotypic difference also leads to enzyme activity differences. Individuals who are homozygous for the ACE D allele have the highest enzyme levels, those homozygous for the ACE I allele have the lowest, and heterozygous individuals have an intermediate level [81]. The D allele of the I/D polymorphism is associated with enhanced ACE levels, leading to higher blood pressure and decreased fibrinolysis during pregnancy, exposing the uteroplacental health at risk [76]. Several studies have indicated the association of ACE I/D polymorphism with Thrombophilia [27, 51], PE [82, 83], and RPL [38] A study by [50] demonstrated the link between fibrinolytic pathway and the RAAS pathway that causes fibrin accumulation. Population-based studies demonstrated that high fibrinogen levels are associated with increased risk of thromboembolic diseases, including enhanced thrombotic risk in placental vessels, which accelerates pregnancy losses. PAI-1 is a vital constituent of the fibrinolysis cascade, and aberrations in PAI-1 levels were associated with placenta-associated obstetric complications, including sporadic and recurrent miscarriages. It was found that the presence of 4G-containing genotypes (4G/4G, 4G/5G) is associated with venous thromboembolism in pregnancy, but not with early abortion [80]. PAI-1 expression is influenced by Ang II plasma levels, which ACE generates. Therefore, ACE polymorphism probably influences implantation [84]. Like thrombophilia genes, the ACE gene also reduces fibrinolysis and restricts bleeding during pregnancy. However, it also increases the risk for other vascular complications, such as PE and abortion [73]. A study in Korean women has revealed the simultaneous association among ACE, AT1R, and AGT gene polymorphisms which influenced the risk of idiopathic RPL [85]. It was suggested that RAAS is central to the pathogenesis of PE [7].

Further, the increase in production of Soluble fms like tyrosine kinase 1 (sFlt1), soluble endoglin (sEng) [62, 72], and AT1-AA [68] leads to the systemic manifestations of PE. ACE plays a critical role in the RAAS, converting angiotensin I to active angiotensin II, a potent vasopressor. ACE has a physiological function in the fibrinolysis pathway as it regulates the concentrations of plasminogen activator Inhibitor I (PAI-1), an essential determinant in controlling the fibrinolytic process. The mutant of the D allele in the ACE gene may compromise placental formation and trophoblastic invasion because of increased PAI-1 expression and concomitant reduced fibrinolytic activity.

## 8. Conclusion

Each year, millions of women are affected by infertility and pregnancy complications such as preclampsia, thrombophilia, recurrent pregnancy loss, preterm birth, and maternal hyperglycemia. These complications are often challenging due to their multitissue and multiorganism nature. Research in the genetic and gene regulatory causes of pregnancy complications has the potential to revolutionize our understanding of healthy and complicated pregnancies. The genetic interactions of the RAAS, immune, coagulation, and fibrinolytic pathways are essential in pregnancy complications or infertility. ACE, PAI-1, VIIa, XIIa, AT1R, AT1AA, and TF are common molecules that can delineate the underlying causes of pregnancy complications. Hence, new studies on these molecules will help avoid complications arise due to unhealthy or nonviable pregnancies. Due to the integration of new evidence and technologies, infertility and pregnancy complications can better be understood to discover underlying biology and potential therapeutic targets.

## References

[1] Vander Borght M., Wyns C. (2018). Fertility and infertility: definition and epidemiology. *Clinical Biochemistry*.

[2] Olooto W. E., Amballi A. A., Banjo T. A. (2012). A review of female infertility; important etiological factors and management. *Journal of Microbiology and Biotechnology Research*.

[3] Simcox L. E., Ormesher L., Tower C., Greer I. A. (2015). Thrombophilia and pregnancy complications. *International Journal of Molecular Sciences*.

[4] Mirabito Colafella K. M., Bovée D. M., Danser A. H. J. (2019). The renin-angiotensin-aldosterone system and its therapeutic targets. *Experimental Eye Research*.

[5] Patel S., Rauf A., Khan H., Abu-Izneid T. (2017). Renin-angiotensin-aldosterone (raas): the ubiquitous system for homeostasis and pathologies. *Biomedicine & Pharmacotherapy*.

[6] Felmeden D. C., Lip G. Y. (2000). The renin-angiotensin-aldosterone system and fibrinolysis. *Journal of the Renin-Angiotensin-Aldosterone System*.

[7] Zhong W. G., Wang Y., Zhu H., Zhao X. (2012). Meta analysis of angiotensin-converting enzyme i/d polymorphism as a risk factor for preeclampsia in Chinese women. *Genetics and Molecular Research*.

[8] Mascarenhas M. N., Flaxman S. R., Boerma T., Vanderpoel S., Stevens G. A. (2012). National, regional, and global trends in infertility prevalence since 1990: a systematic analysis of 277 health surveys. *PLoS Medicine*.

[9] van der Poel S. Z. (2012). Historical walk: the hrp special programme and infertility. *Gynecologic and Obstetric Investigation*.

[10] Naina P., Sharma H. (2021). Prevalence and potential determinants of primary infertility in India: evidence from Indian demographic health survey. *Health*.

[11] Adamson P. C., Krupp K., Freeman A. H., Klausner J. D., Reingold A. L., Madhivanan P. (2011). Prevalence & correlates of primary infertility among young women in Mysore, India. *The Indian Journal of Medical Research*.

[12] Ganguly S., Unisa S. (2010). Trends of infertility and childlessness in India: findings from nfhs data. *Facts, Views & Vision in ObGyn*.

[13] Hart R. J. (2016). Physiological aspects of female fertility: role of the environment, modern lifestyle, and genetics. *Physiological Reviews*.

[14] Legro R. S. (2007). A 27-year-old woman with a diagnosis of polycystic ovary syndrome. *JAMA*.

[15] Merkin S. S., Phy J. L., Sites C. K., Yang D. (2016). Environmental determinants of polycystic ovary syndrome. *Fertility and Sterility*.

[16] Best D., Bhattacharya S. (2015). Obesity and fertility. *The Clinical Investigator*.

[17] Dechanet C., Anahory T., Mathieu Daude J. C. (2011). Effects of cigarette smoking on reproduction. *Human Reproduction Update*.

[18] El Sayed S. L. M., Desoky M. (2019). Effect of lifestyle alteration of pregnant women with mild preeclampsia on maternal and fetal status. *JOJ Nurse Health Care*.

[19] Kohorst M. A., Warad D. M., Nageswara Rao A. A., Rodriguez V. (2018). Obesity, sedentary lifestyle, and video games: the new thrombophilia cocktail in adolescents. *Pediatric Blood & Cancer*.

[20] Ng K. Y. B., Cherian G., Kermack A. J. (2021). Systematic review and meta-analysis of female lifestyle factors and risk of recurrent pregnancy loss. *Scientific Reports*.

[21] Yatsenko S. A., Rajkovic A. (2019). Genetics of human female infertility†. *Biology of Reproduction*.

[22] Zorrilla M., Yatsenko A. N. (2013). The genetics of infertility: current status of the field. *Current Genetic Medicine Reports*.

[23] Al Qahtani N. H., AbdulAzeez S., Almandil N. B. (2021). Whole-genome sequencing reveals exonic variation of asic5 gene results in recurrent pregnancy loss. *Frontiers in Medicine*.

[24] Aslbahar F., Neamatzadeh H., Tabatabaiee R. S. (2018). Association of angiotensin-converting enzyme insertion/deletion polymorphism with recurrent pregnancy loss: a meta-analysis of 26 case-control studies. *Revista Brasileira de Ginecologia e Obstetrícia*.

[25] Sayed-Tabatabaei F. A., Oostra B. A., Isaacs A., van Duijn C. M., Witteman J. C. (2006). Ace polymorphisms. *Circulation Research*.

[26] Carluccio M., Soccio M., De Caterina R. (2001). Aspects of gene polymorphisms in cardiovascular disease: the renin-angiotensin system. *European Journal of Clinical Investigation*.

[27] Mello G., Parretti E., Gensini F. (2003). Maternal-fetal flow, negative events, and preeclampsia: role of ace i/d polymorphism. *Hypertension*.

[28] González-Garrido J. A., García-Sánchez J. R., Tovar-Rodríguez J. M., Olivares-Corichi I. M. (2017). Preeclampsia is associated with *ACE* I/D polymorphism, obesity and oxidative damage in Mexican women. *Pregnancy Hypertens*.

[29] Brosens I., Pijnenborg R., Vercruysse L., Romero R. (2011). The "great obstetrical syndromes" are associated with disorders of deep placentation. *American Journal of Obstetrics and Gynecology*.

[30] Goodman C., Hur J., Goodman C. S., Jeyendran R. S., Coulam C. (2009). Original article: are polymorphisms in the ACE and PAI-1 genes associated with recurrent spontaneous miscarriages?. *American Journal of Reproductive Immunology*.

[31] Coulam C. B., Jeyendran R. S., Fishel L. A., Roussev R. (2006). Multiple thrombophilic gene mutations are risk factors for implantation failure. *Reproductive Biomedicine Online*.

[32] Billings F. T., Balaguer J. M., Yu C. (2012). Comparative effects of angiotensin receptor blockade and ace inhibition on the fibrinolytic and inflammatory responses to cardiopulmonary bypass. *Clinical Pharmacology and Therapeutics*.

[33] Correa-Noronha S. A., Noronha S. M., Alecrim C. (2012). Association of angiotensin-converting enzyme i gene i/d polymorphism with endometrial but not with ovarian cancer. *Gynecological Endocrinology*.

[34] Koika V., Georgopoulos N. A., Piouka A. (2012). Increased frequency of the di genotype of the angiotensin-i converting enzyme and association of the ii genotype with insulin resistance in polycystic ovary syndrome. *European Journal of Endocrinology*.

[35] Buchholz T., Thaler C. J. (2003). Inherited thrombophilia: impact on human reproduction. *American Journal of Reproductive Immunology*.

[36] Aarabi M., Memariani T., Arefi S. (2011). Polymorphisms of plasminogen activator inhibitor-1, angiotensin converting enzyme and coagulation factor xiii genes in patients with recurrent spontaneous abortion. *The Journal of Maternal-Fetal & Neonatal Medicine*.

[37] Kempf-Haber M., Klimek M. (2005). Thrombophilia in pregnancy and its influence on venous thromboembolism and recurrent miscarriages. *Przegla̧d Lekarski*.

[38] Su M. T., Lin S. H., Chen Y. C., Kuo P. L. (2013). Genetic association studies of ace and pai-1 genes in women with recurrent pregnancy loss: a systematic review and meta-analysis. *Thrombosis and Haemostasis*.

[39] Lussana F., Coppens M., Cattaneo M., Middeldorp S. (2012). Pregnancy-related venous thromboembolism: risk and the effect of thromboprophylaxis. *Thrombosis Research*.

[40] Jeddi-Tehrani M., Torabi R., Mohammadzadeh A. (2010). Investigating association of three polymorphisms of coagulation factor xiii and recurrent pregnancy loss. *American Journal of Reproductive Immunology*.

[41] Bennett S. A., Bagot C. N., Arya R. (2012). Pregnancy loss and thrombophilia: the elusive link. *British Journal of Haematology*.

[42] Sammaritano L. R. (2020). Antiphospholipid syndrome. *Best Practice & Research. Clinical Rheumatology*.

[43] Ruiz-Irastorza G., Crowther M., Branch W., Khamashta M. A. (2010). Antiphospholipid syndrome. *Lancet*.

[44] McNamee K., Dawood F., Farquharson R. (2012). Recurrent miscarriage and thrombophilia. *Current Opinion in Obstetrics & Gynecology*.

[45] Baek K. H., Lee E. J., Kim Y. S. (2007). Recurrent pregnancy loss: the key potential mechanisms. *Trends in Molecular Medicine*.

[46] Corbo R. M., Ulizzi L., Piombo L., Scacchi R. (2011). Association of ace i/d polymorphism and recurrent miscarriages in an italian population with a pre-modern reproductive pattern. *Annals of Human Biology*.

[47] Kutluturk I., Karagöz A., Bezgin T. (2014). Relationship between angiotensin i-converting enzyme insertion/deletion gene polymorphism and retinal vein occlusion. *Thrombosis Journal*.

[48] Chen H., Nie S., Lu M. (2015). Association between plasminogen activator inhibitor-1 gene polymorphisms and recurrent pregnancy loss: a systematic review and meta-analysis. *American Journal of Reproductive Immunology*.

[49] Labied S., Blacher S., Carmeliet P. (2011). Transient reduction of placental angiogenesis in pai-1-deficient mice. *Physiological Genomics*.

[50] Kurzawińska G., Barlik M., Drews K. (2016). Coexistence of ace (i/d) and pai-1 (4g/5g) gene variants in recurrent miscarriage in polish population. *Ginekologia Polska*.

[51] Buchholz T., Lohse P., Rogenhofer N., Kosian E., Pihusch R., Thaler C. J. (2003). Polymorphisms in the ace and pai-1 genes are associated with recurrent spontaneous miscarriages. *Human Reproduction*.

[52] Dossenbach-Glaninger A., van Trotsenburg M., Schneider B., Oberkanins C., Hopmeier P. (2008). Ace i/d polymorphism and recurrent first trimester pregnancy loss: interaction with serpine1 4g/5g and f13 val34leu polymorphisms. *British Journal of Haematology*.

[53] Kim J. J., Choi Y. M., Lee S. K. (2014). The PAI-1 4G/5G and ACE I/D polymorphisms and risk of recurrent pregnancy loss: a case–control study. *American Journal of Reproductive Immunology*.

[54] Xia Y., Wen H., Bobst S., Day M. C., Kellems R. E. (2003). Maternal autoantibodies from preeclamptic patients activate angiotensin receptors on human trophoblast cells. *Journal of the Society for Gynecologic Investigation*.

[55] Chou J., Mackman N., Merrill-Skoloff G., Pedersen B., Furie B. C., Furie B. (2004). Hematopoietic cell-derived microparticle tissue factor contributes to fibrin formation during thrombus propagation. *Blood*.

[56] Østerud B. (2010). Tissue factor expression in blood cells. *Thrombosis Research*.

[57] Brill A., Fuchs T. A., Savchenko A. S. (2012). Neutrophil extracellular traps promote deep vein thrombosis in mice. *Journal of Thrombosis and Haemostasis*.

[58] Papayannopoulos V., Zychlinsky A. (2009). Nets: a new strategy for using old weapons. *Trends in Immunology*.

[59] Schulz C., Engelmann B., Massberg S. (2013). Crossroads of coagulation and innate immunity: the case of deep vein thrombosis. *Journal of Thrombosis and Haemostasis*.

[60] Luo D., Szaba F. M., Kummer L. W. (2011). Protective roles for fibrin, tissue factor, plasminogen activator inhibitor-1, and thrombin activatable fibrinolysis inhibitor, but not factor xi, during defense against the gram-negative bacterium yersinia enterocolitica. *Journal of Immunology*.

[61] Kallela J., Jääskeläinen T., Kortelainen E. (2016). The diagnosis of pre-eclampsia using two revised classifications in the Finnish Pre-eclampsia Consortium (FINNPEC) cohort. *BMC pregnancy and childbirth*.

[62] Rana S., Lemoine E., Granger J. P., Karumanchi S. A. (2019). Preeclampsia. *Circulation Research*.

[63] Leung P. S., Tsai S. J., Wallukat G., Leung T. N., Lau T. K. (2001). The upregulation of angiotensin II receptor AT_1_ in human preeclamptic placenta. *Molecular and Cellular Endocrinology*.

[64] Velloso E. P., Vieira R., Cabral A. C., Kalapothakis E., Santos R. A. (2007). Reduced plasma levels of angiotensin-(1-7) and renin activity in preeclamptic patients are associated with the angiotensin i- converting enzyme deletion/deletion genotype. *Brazilian Journal of Medical and Biological Research*.

[65] Ito M., Nakamura T., Yoshimura T., Koyama H., Okamura H. (1992). The blood pressure response to infusions of angiotensin ii during normal pregnancy: relation to plasma angiotensin ii concentration, serum progesterone level, and mean platelet volume. *American Journal of Obstetrics and Gynecology*.

[66] Zhang L., Zhou Y., Wu Q. (2017). Effective prediction of preeclampsia by measuring serum angiotensin ii, urinary angiotensinogen and urinary transforming growth factor *β*1. *Experimental and Therapeutic Medicine*.

[67] Rahimi Z., Rahimi Z., Mozafari H., Parsian A. (2013). Preeclampsia and angiotensin converting enzyme (ace) i/d and angiotensin ii type-1 receptor (at1r) a1166c polymorphisms: association with ace i/d polymorphism. *Journal of the Renin-Angiotensin-Aldosterone System*.

[68] Campbell N., LaMarca B., Cunningham M. W. (2018). The role of agonistic autoantibodies to the angiotensin ii type 1 receptor (at1-aa) in pathophysiology of preeclampsia. *Current Pharmaceutical Biotechnology*.

[69] Jensen F., Wallukat G., Herse F. (2012). Cd19+cd5+ cells as indicators of preeclampsia. *Hypertension*.

[70] Herse F., LaMarca B. (2013). Angiotensin ii type 1 receptor autoantibody (at1-aa)-mediated pregnancy hypertension. *American Journal of Reproductive Immunology*.

[71] Harmon A. C., Cornelius D. C., Amaral L. M. (2016). The role of inflammation in the pathology of preeclampsia. *Clinical Science*.

[72] Zhou C. C., Ahmad S., Mi T. (2007). Angiotensin ii induces soluble fms-like tyrosine kinase-1 release via calcineurin signaling pathway in pregnancy. *Circulation Research*.

[73] Yang C., Fangfang W., Jie L. (2012). Angiotensin-converting enzyme insertion/deletion (i/d) polymorphisms and recurrent pregnancy loss: a meta-analysis. *Journal of Assisted Reproduction and Genetics*.

[74] Khan S., Dar S. A., Mandal R. K. (2018). Angiotensin-converting enzyme gene i/d polymorphism is associated with systemic lupus erythematosus susceptibility: an updated meta-analysis and trial sequential analysis. *Frontiers in Physiology*.

[75] Zhao J., Qin X., Li S., Zeng Z. (2014). Association between the *ACE* I/D polymorphism and risk of ischemic stroke: an updated meta-analysis of 47,026 subjects from 105 case -control studies. *Journal of the Neurological Sciences*.

[76] Gintoni I., Adamopoulou M., Yapijakis C. (2021). The angiotensin-converting enzyme insertion/deletion polymorphism as a common risk factor for major pregnancy complications. *In Vivo*.

[77] Li X., Liu Y., Zhang R., Tan J., Chen L., Liu Y. (2015). Meta-analysis of the association between plasminogen activator inhibitor-1 4g/5g polymorphism and recurrent pregnancy loss. *Medical Science Monitor*.

[78] Gumus E. (2018). The powerful association of angiotensin-converting enzyme insertion/deletion polymorphism and idiopathic recurrent pregnancy loss. *Ginekologia Polska*.

[79] Findeklee S., Costa S. D., Tchaikovski S. N. (2015). Thrombophilia and hellp syndrome in pregnancy - case report and overview of the literature. *Zeitschrift für Geburtshilfe und Neonatologie*.

[80] Magdoud K., Herbepin V. G., Touraine R., Almawi W. Y., Mahjoub T. (2013). Plasminogen activator inhibitor 1 4G/5G and −844G/A variants in idiopathic recurrent pregnancy loss. *American Journal of Reproductive Immunology*.

[81] Wang Z., Wang P., Wang X. (2013). Significant association between angiotensin-converting enzyme gene insertion/deletion polymorphism and risk of recurrent miscarriage: a systematic review and meta-analysis. *Metabolism*.

[82] Procopciuc L. M., Nemeti G., Buzdugan E., Iancu M., Stamatian F., Caracostea G. (2019). Renin-angiotensin system gene variants and risk of early- and late-onset preeclampsia: a single center case-control study. *Pregnancy Hypertension*.

[83] Shaheen G., Sajid S., Razak S. (2019). Role of *ACE* I/D polymorphism in pathological assessment of preeclampsia in Pakistan. *Molecular Genetics & Genomic Medicine*.

[84] Zhang S., Wang J., Wang B., Ping Y., Ma X. (2011). Strong association between angiotensin i-converting enzyme i/d polymorphism and unexplained recurrent miscarriage of chinese women--a case-control study. *Reproductive Sciences*.

[85] Choi Y. S., Kwon H., Kim J. H. (2011). Haplotype-based association of ace i/d, at1r 1166a>c, and agt m235t polymorphisms in renin-angiotensin-aldosterone system genes in korean women with idiopathic recurrent spontaneous abortions. *European Journal of Obstetrics, Gynecology, and Reproductive Biology*.

